# Study on the association between sleep disorders versus oral health related variables

**DOI:** 10.4317/medoral.24096

**Published:** 2020-08-27

**Authors:** Duziene Pereira, Patrícia Progiante, Marcos Pattussi, Patrícia Grossi, Márcio Grossi

**Affiliations:** 1 DDS, MS, PhD, Post-Graduate Program in Dentistry (Prosthodontics), School of Health and Life Sciences, Pontifical Catholic University of Rio Grande do Sul (PUCRS), Brazil; 2 DDS, MS, PhD, Post-Doctoral Fellow University of São Paulo (USP) Faculty of Dentistry, Av. Lineu Prestes 2227, São Paulo, SP, 05508-000, Brazil; 3 DDS, MSc, PhD, Post-Graduate Program in Public Health, Vale do Rio dos Sinos University (UNISINOS), Brazil; 4 BSW, MSW, PhD, Professor, Post-Graduate Program in Social Work, School of Humanities, Pontifical Catholic University of Rio Grande do Sul (PUCRS), Brazil

## Abstract

**Background:**

To study the association between sleep quality and oral health related variables, which still have conflicts in the literature.

**Material and Methods:**

This was a population-based case-control study between subjects with versus without sleep disorders from the Brazilian Public Health System (SUS), city of Maringá (N=1,643). Subjects answered self-reported questionnaires: a) Research Diagnostic Criteria for Temporomandibular Disorders (RDC/TMD), b) Sleep Assessment Questionnaire (SAQ) and c) North York Dental Health Survey (NYDHS).

**Results:**

No significant difference was found for gender, marital status, or income; however, non-Caucasians, people with lower levels of education, and those between 20 to 50 years old had worse scores of sleep disorders in the SAQ. Self-perceived oral health, masticatory capacity to eat foods, and gingival bleeding was significantly worse among subjects with self-reported sleep disorders. Self-reported tooth loss, edentulism and use of removable partial dentures (with clasps) or complete dentures showed no significant difference between groups. Self-reported sleep disorder subjects presented significantly higher prevalence of both self-reported tooth and TMJ pain.

**Conclusions:**

It can be concluded that individuals with self-reported sleep disorders presented worse self-perceived oral health for most studied variables.

** Key words:**Oral health, case control study, sleep; review, gingivitis, periodontitis, tooth loss.

## Introduction

Sleep is comprised by different stages, and it is composed of two distinct and alternating phases: a) NREM or non-rapid eye movement, which is also known as synchronized sleep, quiet, and slow wave sleep; and b) REM or rapid eye movement, which is active, fast, parasomnia, or paradoxical sleep (1).

Sleep is a behavioral and physiological state, generally resistant to non-mental external stimuli; however, in case these external stimuli become too strong to be ignored, a sleep disorder can be developed, which is a common factor in the worsening of the quality of life (2). Poor sleep quality may lead to fatigue, depression, loss of concentration, increase in irritability, and anxiety (3-5). The lack of sleep can also lead to pain and an increase in pain sensitivity (6). In patients with acute pain, generally, pain is preceded by poor sleep at night. Patients with chronic pain show a loop system, in which poor sleep at night can be followed by pain in the following day, and high levels of pain during the day is frequently followed by poor sleep at night (3,6).

Poor oral and general health may also influence and be influenced by sleep quality (5). Sleep disorders (i.e., sleep breathing disorders, snoring and oral breathing during sleep) may affect oral health and be associated to gingival inflammation (5,7). Some publications associated sleep apnea and sleep disorders with periodontitis (8,9,10). Research suggests that complete edentulism and sleeping without the prosthesis also favor sleep breathing disorders (11,12). Some studies demonstrated that obstructive sleep apnea (OSA) might be detected by cephalometric evaluation in edentulous patients and those who use complete dentures (13,14).

The objective of this article was both to study self-reported sleep disorders and their associated social and economic factors in an adult population and to test the association between self-reported sleep disorders with self-perceived oral health related variables.

## Material and Methods

- Population, Inclusion and Exclusion Criteria

This population-based case-control study was comprised by subjects with ages between 20 to 65 years, registered users of the Brazilian Public Health System (SUS) in the city of Maringá, state of Paraná. The Maringá SUS database has 132,620 people from the general population, with an age range from 20 to 65 years of age. The data was collected between August 2011 and March 2012, and it was obtained from a previous population cross sectional survey in the city of Maringá, Brazil; using structured and valid questionnaires, and further detail can be found elsewhere (15).

Only those registered as active users of the Maringá SUS database were included to prevent duplicity of insertion, or inclusion of subjects who moved or passed away. Patients making use of anxiolytics, anticonvulsants, or opioid analgesics were excluded as well as those with any kind of systemic diseases, chronic pain conditions, or psychological disorders in the medical history (15). These excluding factors were selected, because they might have created confounders and affected the reliability of the questionnaires used, considering that they might have interfered in the diagnosis of sleep and/or pain disorders (16). All self-completing questionnaires were given to patients by the same researcher.

- Research Questionnaires

For the data collection, only structured and validated questionnaires were used, which included demographic, socio-economic, oral health, and sleep related variables:

a) The Axis II of the Research Diagnostic Criteria for Temporomandibular Disorders (RDC/TMD) assesses pain impairment and pain related psychosocial variables (i.e., depression and somatization) and questions regarding to oral and general health (16). In this study, only questions assessing socio-economic factors related to oral health were used.

b) The Sleep Assessment Questionnaire (SAQ) is a self-completing questionnaire with 17 questions, where each answer is scored from 0 to 4 (i.e., never, rarely, sometimes, often, always, and don’t know), giving a maximum score of 68. The higher the score, the worse is the sleep disorder. The cut-off point is 16 (i.e., 16 or higher is positive for sleep disorders), because it has been shown to have the highest sensitivity (0.74) and specificity (0.80) when compared to polysomnography for different sleep disorders (i.e., non-restorative sleep and insomnia) (17). Subjects with a positive sleep disorder test result in the SAQ became the test group, while the ones with a negative test result, became the control group.

c) The North York Dental Health Survey (NYDHS) was developed for epidemiological studies on the patients’ perception of their oral health, including TMD signs and symptoms, bleeding gums, missing teeth, and capacity to chew, swallow and talk. This questionnaire has been successfully used for public health planning in Canada (18).

- Sample size calculation and statistical analysis

The final calculated number of the population-based case-control study yielded a total of 1,365 individuals, using the following parameters: prevalence of TMD = 5% in the non-exposed group and 10% in the exposed group, 1:8 ratio between exposed versus non-exposed, type I error = 5%, type II error = 20%, statistical power = 80%, and power to detect a risk ratio = 2 with 95% confidence interval. An additional 30% was added to compensate for losses and missing values, giving a final sample size of 1,775 individuals (15). Data analysis was conducted using SPSS 20.0 and STATA 11.0. Univariate analysis was performed using Pearson’s Chi Square and Linear-by-Linear Association for categorical data, while Student’s t-test was used for continuous data. In categorical data, crude and adjusted logistic regression (LR) analysis was used to calculate the risk factor of sleep disorders in developing oral health related problems.

## Results

The final sample was comprised by 1,643 subjects due to a high recruitment rate (92.56%). Patients were predominantly women (65.9%), young adults (84.7%), between 20 to 49 years of age (32.7 ± 10.3), married or single (90.6%), with high school or post-secondary education (79.9%).

Table 1 shows the social and demographic data from the RDC/TMD Axis II of subjects with sleep disorders versus those without measured by the SAQ. It can be observed that more than half (56.8%) of the studied population had sleep disorders. No statistically significant difference between genders, marital status, and family income was found. Significant differences were found in the age distribution, where the majority of those with sleep disorders were predominantly between the ages of 20 to 50 years. Besides that, significant differences were also found in ethnicity, which showed a higher prevalence of sleep disorders in non-Caucasians against Caucasians (32.9% versus 25.9%, respectively), and level of education, where the prevalence of sleep disorders was higher in those with incomplete high school/complete elementary school (24.7% versus 14.1%, respectively).

Self-perceived overall oral health and masticatory capacity related variables were measured by the North York Dental Health Survey, and the results are shown in Table 2, Table 3. In Table 2, regarding self-perception of oral health and masticatory capacity, it can be observed that 85.6% of subjects without sleep disorders classified their oral health as excellent, very good or good, against 71.8% of those with sleep disorders (<0.001), with an increase in the chance of developing sleep disorders in more than two times (adjusted OR=2.1). When questioned about the need to visit a dentist, the majority of sleep disorder subjects reported significantly higher need as compared to those without it, 86.8% versus 78.0%, respectively.

**Table 1:**
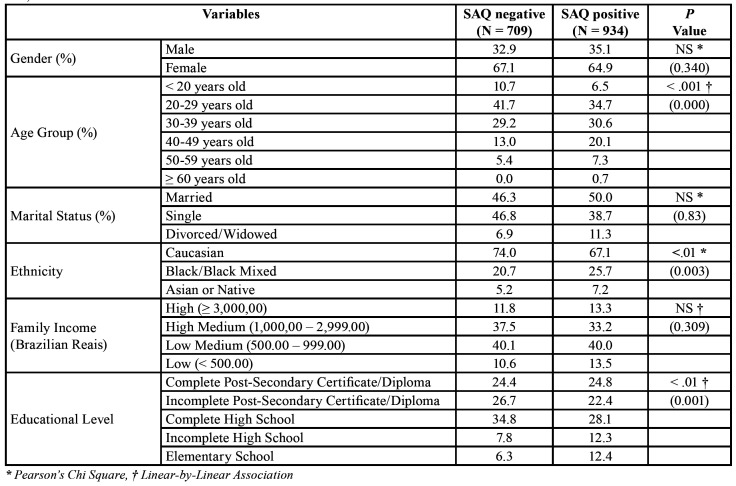
Social and demographic description from the Research Diagnostic Criteria for Temporomandibular Disorders (RDC/TMD) Axis II between subjects with sleep disorders measured by the SAQ cases (Sleep Assessment Questionnaire, global score 16 or higher) versus controls (global score less than 16) of our sample extracted from the population of the City of Maringá users of the Brazilian Public Health System (SUS), N=1,643.

**Table 2:**
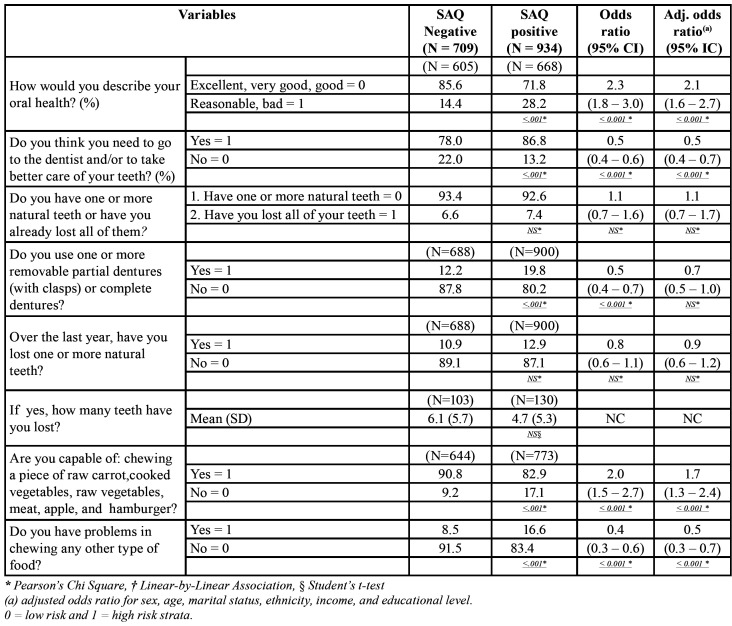
Prevalence of the North York Dental Health Survey (NYDHS) for self-perceived oral health, number of teeth and chewing ability between patients with sleep disorders measured by the SAQ (Sleep Assessment Questionnaire) global score versus controls in our sample extracted from the population of the City of Maringá, users of the Brazilian Public Health System (SUS), N=1,643. SAQ positive = global score equal or greater than 16.

In respect to edentulism, there was a very low and non-significant absolute percent difference (0.8%) between sleep versus non-sleep disorder groups. Coherently, despite sleep disorder subjects presented a significantly higher percentage of wearers of removable partial dentures (with clasps) or complete dentures as compared with those without sleep disorders (19.8% versus 12.2%), the adjusted OR was non-significant. In addition, the average number of missing teeth was also non-significant between sleep versus non-sleep disorder subjects (4.7 versus 6.1, respectively).

Regarding self-perceived masticatory capacity, the vast majority of subjects in both groups felt they were able to chew soft or hard food (i.e., raw carrot, cooked vegeTables, raw vegeTables, meat, apple, and hamburger); however, the group with sleep disorders reported significantly more chewing problems in eating these foods than the those without them (17.1% versus 9.2%), increasing the risk of developing chewing problems in 70% (adjusted OR=1.7). Similarly, sleep disorder subjects also reported significantly more problems in eating other types of foods not listed above (16.6% versus 8.5%).

In Table 3, it can be observed that subjects with sleep disorders have shown more TMD and tooth related pain as well as self-reported gingival bleeding problems than those without them. Subjects in the sleep disorder group reported significantly more tooth pain than controls (38.3% versus 25.1%), increasing in almost two times the risk of developing it (adjusted OR=1.9). Similarly, subjects with sleep disorders also reported significantly more pain in front of the ear, face or cheeks as compared to controls (31.6% versus 10.6%), increasing in four times the risk of developing pain symptoms related to TMD (adjusted OR=4.0).

**Table 3:**
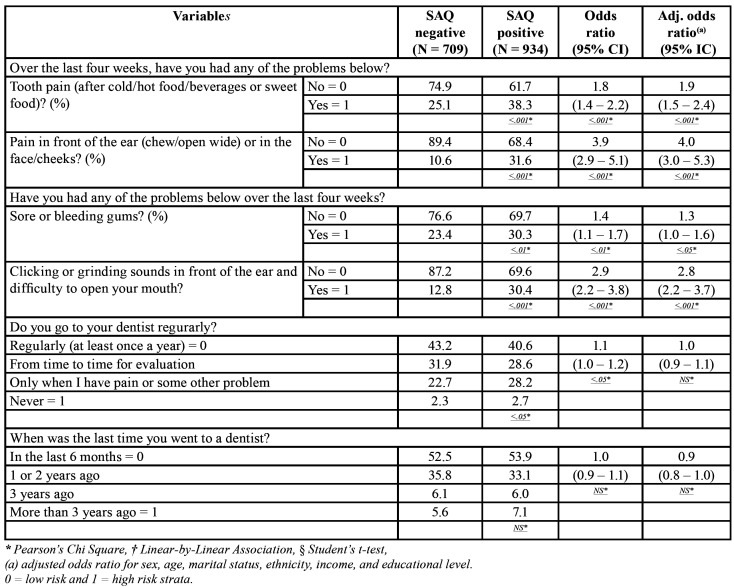
Prevalence of the North York Dental Health Survey (NYDHS) for self-perceived caries, periodontal disease, oral lesions, and TMJ arthritis between patients with sleep disorders measured by the SAQ (Sleep Assessment Questionnaire) global score versus controls in our sample extracted from the population of the City of Maringá, users of the Brazilian Public Health System (SUS), N=1,643. SAQ positive = global score equal or greater than 16.

Similarly and coherently, clicking and grinding sounds in front of the ear and difficulty to open the mouth were also significantly more prevalent in the sleep disorder group versus controls (30.4% versus 12.8%), increasing in almost three times the risk of developing TMJ sounds (adjusted OR=2.9).

Regarding the subject’s periodontal health, it could be observed that subjects with sleep disorders reported a significantly higher percentage of sore or bleeding gums when compared with those without sleep disorders (30.3%versus 23.4%), increasing in 30% the risk of developing bleeding gums (adjusted OR=1.3). However, when subjects were asked about the frequency of visits to a dentist, significant difference was either not found, or it did not continue to be significant after controlling for confounders in the LR analysis, showing that individuals with sleep disorders despite reporting worse oral health, do not seek treatment for that.

Our adjusted LR analysis for all variables measured in Table 2 and Table 3 was controlled for sex, age, marital status, ethnicity, income, and educational level. These confounders did not change substantially the absolute crude OR described in Table 2 and Table 3 in most variables, with the exception of frequency of attending a dentist and the use of removable partial dentures (with clasps) or complete dentures, where a significant crude OR was changed to a non-significant adjusted OR.

## Discussion

Our data showed that individuals with non-Caucasian ethnicity, with lower level of education, and in reproductive age (i.e., between 20 to 50 years old) reported more sleep disorders (Table 1). This might be due to the higher social and stress levels and with greater probability of developing sleep disorders in this particular group, which is in agreement with the reports of the literature (19). In contrast, no significant difference could be found in this study regarding gender, marital status, and income and the presence of self-reported sleep disorders. This contrasts with the literature, where studies have shown higher prevalence of insomnia among women, especially over the age of 40 years old, as well as in other sleep disorders (19,20). This might be explained, because in this investigation: age, marital status, ethnicity, income, and educational level were controlled in the LR analysis; in addition, other systemic conditions were controlled during the inclusion and exclusion criteria, and they all influence sleep disorders.

From the data in Table 2, it can be interpreted that patients with reported sleep disorders reported substantially more oral health problems than those without them. Similarly to the literature, patients who reported their oral health as excellent, very good or good presented lower sleep disorders scores, which reduces the probability of presenting poor sleep, fatigue and low vitality (21,22). Acar *et al*, showed significant positive correlation between the duration of snoring complaints with dental and oral health reports (23).

In this study, there was a higher proportion of individuals with total edentulism between the sleep disorder group as compared to the control group (7.4% versus 6.6%, respectively); and they also had higher proportion of natural tooth loss in the last year. Coherently, the group with sleep disorders also significantly reported more chewing problems in eating soft or hard food. This is also in agreement with the literature, where one study observed that a group of patients with sleep disorders presented reduced masticatory function due to insufficient number of teeth (7). Other studies have also shown that tooth loss is an oral problem that might be associated with sleep disorders, such as snoring and obstructive sleep apnea (11,12). It has been suggested that this happens because tooth loss produces anatomic alterations that might reduce the size and function of the upper airway space (7). However, in one study, a low index of tooth loss and satisfactory masticatory function was observed in more than 80% of the individuals in both users and non-users of CPAP/BiPAP (continuous or bi-level positive airway pressure) (24). Therefore, it is still unproved if tooth loss induces sleep disorders; however, tooth loss is related to masticatory capacity.

Higher proportion was found in this investigation regarding the use of removable partial dentures (with clasps) or complete dentures between those with sleep disorders versus those without them (19.8% versus 12.2%, respectively), but the results were non-significant after controlling for confounders. The literature has been contradictory regarding the role of complete denture use and sleep quality. Emami *et al* found no significant difference in the sleep quality or in the perception of daytime sleepiness in edentulous elderly between those who wore their dentures at night and those who did not using self-report questionnaires for sleep quality and daytime sleepiness (Pittsburgh Sleep Quality Index – PSQI and Epworth Sleepiness Scale - ESS) (4). On the other hand, Mattia *et al* evaluated the sleep quality and sleep apnea using objective (ApneaLink Plus 9.0, BiteStrip) and subjective methods (SAQ, PSQI -Brazilian version, and the ESS – Brazilian version) in patients users of mucosa-supported superior complete dentures against lower implant-retained complete dentures at night (25). The authors showed that the objective measurements of sleep bruxism activity and the respiratory disturbance index showed significant reduction (i.e., improvement) when patients did not wear the maxillary complete dentures. The subjective measurements of sleep quality and self-reports of SB activity showed no significant differences between wearing and not wearing maxillary complete dentures at night (25). Therefore, more studies using objective measures of sleep disorders in a sleep laboratory should be performed to clarify this topic.

From Table 3, it can be observed in this investigation that patients with sleep disorders showed higher levels of tooth pain and pain in front of the year suggestive of TMD. In a recent population study, self-reported sleep disorders using the SAQ were correlated with TMD, and the authors found that individuals with TMD presented higher prevalence of sleep disorders in all RDC/TMD axis I sub-groups (i.e., myofascial pain and arthralgia/osteoarthritis/osteoarthrosis), with the exception of disc interference disorders due to the lower pain intensity in this sub-group (26). Similarly, the best predictors of musculoskeletal pain are: individuals with 30 to 39 years of age, with high body mass index, with low socio-economic level, without physical activity, with non-restorative sleep, with daytime sleepiness, with anxiety and depression symptoms, and with low quality of life (27). These data in combination, might lead to the conclusion that chronic pain might be associated with poor sleep, and this association might develop a loop, where one disease might increase the other (1,3).

In this study, subjects with sleep disorders presented higher percentages of self-reported gingival bleeding when compared to those without sleep disorders. This is in agreement with the literature, where Individuals with self-reported sleep disorders have shown higher levels of gingival inflammation than those without, which might increase the risk of periodontal disease (7,9,28). Other authors also confirmed the relationship between the severity of periodontitis and the amount of dental plaque in subjects with obstructive sleep apnea (29,30). Nizam *et al* found similar results, despite finding non-significant differences in the periodontal clinical measurements between patients with obstructive sleep apnea versus those without it (8). The possible reasons for these findings is the fact that sleep disorders change the immune system and induce a systemic inflammation (9). The salivary levels of IL-6 and IL-33, which regulates the cell recruitment in the transition from acute to chronic inflammation, are susceptible to increase in patients with obstructive sleep apnea, regardless of the severity of the periodontal disease; in addition, their concentration might present a role in the pathogenesis of periodontal disease in these patients (8).

## Conclusions

Based on the findings in this investigation, it can be concluded that subjects with self-assessed poor oral health related variables (i.e., tooth and TMD pain, decreased masticatory capacity in chewing soft or hard food, gingival bleeding, and need for oral care from a dentist) also reported significantly more sleep disorders. However, no significant difference was found for self-assessed edentulism, tooth loss, and use of removable partial dentures (with clasps) or complete dentures.

## References

[1] Lavigne GJ, Sessle BJ (2016). The neurobiology of orofacial pain and sleep and their interactions. J Dent Res.

[2] Baldwin CM, Ervin AM, Mays MZ, Robbins J, Shafazand S, Walsleben J (2010). Sleep disturbances, quality of life, and ethnicity: the sleep heart health study. J Clin Sleep Med.

[3] Brousseau M, Manzini C, Thie N, Lavigne G (2003). Understanding and managing the interaction between sleep and pain: an update for the dentist. J Can Dent Assoc.

[4] Emami E, Lavigne G, de Grandmont P, Rompré PH, Feine JS (2012). Perceived sleep quality among edentulous elders. Gerodontology.

[5] Huynh NT, Emami E, Helman JI, Chervin RD (2014). Interactions between sleep disorders and oral diseases. Oral Dis.

[6] Lopez-Jornet P, Lucero-Berdugo M, Castillo-Felipe C, Zamora Lavella C, Ferrandez-Pujante A, Pons-Fuster A (2015). Assessment of self-reported sleep disturbance and psychological status in patients with burning mouth syndrome. J Eur Acad Dermatol Venereol.

[7] Carra MC, Schmitt A, Thomas F, Danchin N, Pannier B, Bouchard P (2017). Sleep disorders and oral health: a cross-sectional study. Clin Oral Investig.

[8] Nizam N, Basoglu OK, Tasbakan MS, Nalbantsoy A, Buduneli N (2014). Salivary cytokines and the association between obstructive sleep apnea syndrome and periodontal disease. J Periodontol.

[9] Lee CF, Lin MC, Lin CL, Yen CM, Lin KY, Chang YJ (2014). Non-apnea sleep disorder increases the risk of periodontal disease: a retrospective population-based cohort study. J Periodontol.

[10] Hajishengallis G (2015). Periodontitis: from microbial immune subversion to systemic inflammation. Nat Rev Immunol.

[11] Bucca C, Cicolin A, Brussino L, Arienti A, Graziano A, Erovigni F (2006). Tooth loss and obstructive sleep apnoea. Respir Res.

[12] Almeida FR, Furuyama RJ, Chaccur DC, Lowe AA, Chen H, Bittencourt LR (2012). Complete denture wear during sleep in elderly sleep apnea patients-a preliminary study. Sleep Breath.

[13] Oksayan R, Sökücü O, Uyar M, Topçuoğlu T (2015). Effects of edentulism in obstructive sleep apnea syndrome. Niger J Clin Pract.

[14] Gupta P, Thombare R, Pakhan AJ, Singhal S (2011). Cephalometric evaluation of the effect of complete dentures on retropharyngeal space and its effect on spirometric values in altered vertical dimension. ISRN Dentistry.

[15] Progiante PS, Pattussi MP, Lawrence HP, Goya S, Grossi PK, Grossi ML (2015). Prevalence of temporomandibular disorders in an adult Brazilian community population using the research diagnosticcriteria (axes I and II) for temporomandibular disorders (the Maringá study). Int J Prosthodont.

[16] Dworkin SF, Leresche L (1992). Research diagnostic criteria for temporomandibular disorders: review, criteria, examinations and specifications, critique. J Craniomandib Disord.

[17] Unger ER, Nisenbaum R, Moldofsky H, Cesta A, Sammut C, Reyes M (2004). Sleep assessment in a population-based study of chronic fatigue syndrome. BMC Neurol.

[18] Hawkins RJ, Main PA, Locker D (1998). Oral health status and treatment needs of Canadian adults aged 85 years and over. Spec Care Dentist.

[19] Chen Y, Kartsonaki C, Clarke R, Guo Y, Yu C, Bian Z (2018). Characteristics and correlates of sleep duration, daytime napping, snoring and insomnia symptoms among 0.5 million Chinese men and women. Sleep Med.

[20] Guarnieri B (2019). Sleep disorders and cognitive alterations in women. Maturitas.

[21] Koyama S, Aida J, Cable N, Tsuboya T, Matsuyama Y, Sato Y (2018). Sleep duration and remaining teeth among older people. Sleep Medicine.

[22] Asawa K, Sen N, Bhat N, Tak M, Sultane P, Mandal A (2017). Influence of sleep disturbance, fatigue, vitality on oral health and academic performance in Indian dental students. Clujul Med.

[23] Acar M, Türkcan I, Özdas T, Bal C, Cingi C (2015). Obstructive sleep apnoea syndrome does not negatively affect oral and dental health. J Laryngol Otol.

[24] Carra MC, Thomas F, Schimitt A, Pannier B, Danchin N, Bouchard Ph (2016). Oral health in patients treated by positive airway pressure for obstructive sleep apnea: a population-based case-control study. Sleep Breath.

[25] Mattia PRC, Selaimen CMP, Teixeira ER, Fagondes SC, Grossi ML (2018). The effects of sleeping with or without prostheses on sleep quality, sleep bruxism, and signs of obstructive sleep apnea syndrome: a pilot study. Int J Prosthodont.

[26] Rehm DDS, Progiante PS, Pattussi MP, Pellizzer EP, Grossi PK, Grossi ML (2020). Sleep disorders in patients with temporomandibular disorders (TMD) in an adult population-based cross-sectional survey in southern Brazil. Int J Prosthodont.

[27] Roizenblatt S, Souza AL, Palombini L, Godoy LM, Tufik S, Bittencourt LRA (2015). Musculoskeletal pain as a marker of health quality. Findings from the epidemiological sleep study among the adult population of São Paulo city. PLoS One.

[28] Al-Jewair TS, Al-Jasser R, Almas K (2015). Periodontitis and obstructive sleep apnea's bidirectional relationship: a systematic review and meta-analysis. Sleep Breath.

[29] Gunaratman K, Taylor B, Curtis B, Cistulli B (2009). Obstructive sleep apnoea and periodontitis: a novel association?. Sleep Breath.

[30] Gamsiz-Isik H, Kiyan E, Bingol Z, Baser U, Ademoglu E, Yalcin F (2017). Does obstructive sleep apnea increase the risk for periodontal disease? A case-control study. J Periodontol.

